# Myelodysplastic syndrome with clonal karyotype evolution associated with trisomy 8 and *ASXL1* mutation in well‐controlled HIV patient: Case report and literature review

**DOI:** 10.1002/jha2.14

**Published:** 2020-06-02

**Authors:** Daniela Palheiro Mendes‐de‐Almeida, Viviane Lamim Lovatel, Filipe Vicente dos Santos‐Bueno, Elaiza Almeida Antônio de Kós, Francianne Gomes Andrade, Marcia Trindade Schramm, Estevão Portela Nunes, Beatriz Gilda J. Grinsztejn, Maria S Pombo‐de‐Oliveira, Teresa de Souza Fernandez

**Affiliations:** ^1^ Hematology Department Evandro Chagas National Institute of Infectious Diseases Oswaldo Cruz Foundation (FIOCRUZ) Rio de Janeiro Brazil; ^2^ Pediatric Haematology‐Oncology Program Research Centre National Institute of Cancer (INCA) Rio de Janeiro Brazil; ^3^ Division of Cancer Epidemiology and Clinical Research Department of Pediatrics University of Minnesota Minneapolis Minnesota; ^4^ Cytogenetic Department Bone Marrow Transplantation Centre (CEMO) National Institute of Cancer (INCA) Rio de Janeiro Brazil; ^5^ Hematology Department National Institute of Cancer (INCA) Rio de Janeiro Brazil; ^6^ Laboratory of Clinical Research on STD/AIDS Evandro Chagas National Institute of Infectious Disease (INI) Oswaldo Cruz Foundation (FIOCRUZ) Rio de Janeiro Brazil

To the Editor

Nonspecific myelodysplastic features (MDF) due to hematopoietic effects of the human immunodeficiency virus (HIV) were frequent before the antiretroviral therapy (ART) era. Pronounced hypocellularity, plasmacytosis, and eosinophilia were observed and are referred to as HIV myelopathy [1, 2]. These MDF alterations generally resolve with the institution of ART. They should be separated from myelodysplastic syndrome (MDS), a heterogeneous group of myeloid clonal diseases. Myelodysplastic syndromes can have a primary cause, also called *de novo* MDS, or secondary (sMDS), both with the risk of progression to acute myeloid leukemia (AML). Some case reports and small case series of MDS in well‐controlled HIV patients were described [3, 4, 5, 6]. Herein, a new case of a long‐stand well‐controlled HIV infected patient with MDS is reported. The MDS status evolved with clonal karyotype associated with trisomy 8 and *ASXL1* mutation.

A 61‐year‐old black woman was diagnosed with HIV in 2002, and ART—Zidovudine, Lamivudine (3TC), and Efavirenz—was initiated. She remained on this treatment for 16 years with long‐term suppressed viral load, and CD4 cell counts >600 cells/mm^3^. In January 2018, she presented with progressive anemia and mental depression and the scheme was switched to Tenofovir, 3TC, and Dolutegravir. In September 2018, she developed severe anemia and thrombocytopenia (hemoglobin [Hb] 4.3 g/dL, leucocyte 8.7 × 10^9^/L, neutrophils 2.2 × 10^9^/L, and platelets 45 × 10^9^/L, reticulocyte 0.13%, and serum ferritin 1225 μg/L) with red blood cell transfusion dependency. The polymerase chain reaction for Parvovirus B19 was negative on the blood sample. Bone marrow (BM) aspiration and biopsy were performed. The morphology disclosed a BM hypercellular with multilineage dysplasia (80%) and blast cells (5%). The histology was characterized by erythroid reduction and granulocytic hyperplasia with increased precursor cells and abnormal localization of immature precursors (ALIP). No acid‐fast or fungal microorganisms were observed. The G‐banding of BM cells identified the karyotype: 47,XX,+8[5]/46,XX[9] (Figure 1A), and the fluorescence *in situ* hybridization (FISH) analysis confirmed the trisomy 8 (Figure 1B). *ASXL1* and *DNMT3A* somatic mutations were tested by Sanger sequencing [7, 8]. *DNMT3A* was wild type, and *ASXL1* mutation in exon 12 (c.1772dup; p.Y591*A) was detected (Figure 1C). Additionally, two nonpathogenic single nucleotide polymorphisms were identified in exon 12 of *ASXL1* (c.3973C>A p.L1325F; c.1965 C>T p.T655T) (Figure 1C). The patient was diagnosed with refractory anemia with excess of blasts 1 (MDS‐EB‐1) and at very high risk according to the revised International Prognostic Scoring System [9, 10]. The patient was under supportive treatment care with ART, red blood cell and platelet transfusions, erythropoietin, and one cycle of Azacitidine (75 mg/m^2^/day for 7 days) without improvement. Her renal function was preserved, and 7 months later the karyotype of the BM cells demonstrated a clonal evolution: 47,XX,+8[7]/48,XX,+8,+21[6]/49,XX,+8,+10,+21[9]/46,XX[4] (Figure 1D). FISH analysis using the probe LSI RUNX1 break apart (*RUNX1* 21q22 spectrum green/RUNX1T1 8q21 spectrum orange) identified cells with both trisomy 8 and trisomy 21 (Figure 1E). BM analysis maintained the MDS‐EB‐1 pattern, showing a pleomorphic and hyperplastic bone marrow, absence of megakaryocytic sector, erythroid aplasia, hyperplasia myeloid with dysplasia, and 5% of blasts. The biopsy of bone marrow depicted ALIP and erythroid aplasia. Despite all efforts, the hematological parameters disclosed low Hb level (3.5 g/dL), increasing white blood cell count (19.8 × 10^9^/L), and deficient platelet levels (7 × 10^9^/L) with circulating myeloblasts (3%) and the patient expired in July 2019.

**FIGURE 1:**
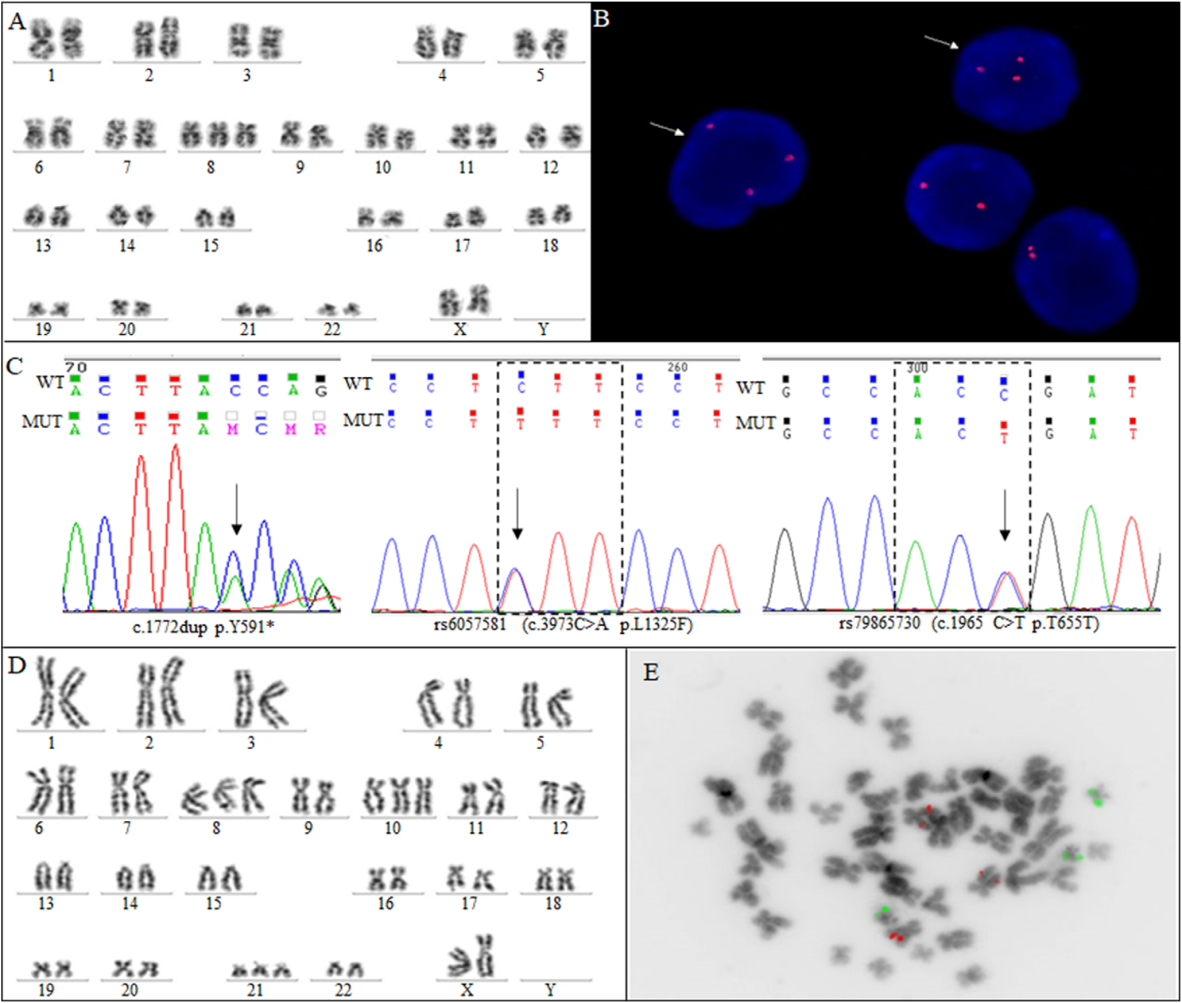
Cytogenetic and molecular alterations in a well‐controlled HIV patient with MDS. **A**, G‐banded showing the karyotype 47,XX,+8. **B**, FISH analysis using LSI MYC SpectrumOrange, red signal, with interphase nuclei counterstain with DAPI, Vysis. The arrows show an extra copy of the c‐myc gene, showing the gain of chromosome 8. **C**, Electropherogram of *ASXL1* sequencing showing a frameshift mutation in exon 12 (c.1772dup; p.Y591*A) and two single nucleotide polymorphisms identified in exon 12 of *ASXL1* (c.3973C>A p.L1325F; c.1965 C>T p.T655T). Arrows indicate the point of the mutation and polymorphisms. **D**, G‐banded showing the karyotype 49,XX,+8,+10,+21. **E**, FISH analysis using the probe LSI RUNX1 break apart (RUNX1 21q2 spectrum green/RUNX1T1 8q21spectrum orange) showing a metaphase with three red signals demonstrating the trisomy 8 and three green signals showing the trisomy 21

A broad scientific review disclosed that only 23 cases of well‐controlled HIV patients with MDS were described (Table 1). HIV portends a poor prognosis in MDS and patients have an increased prevalence of complex karyotypes characterized by monosomy 7 or deletion 7q at cytogenetic level, whereas at the molecular level, the most frequent alterations are somatic mutations in the *ASXL1* exons11‐ 12, *P53*, and *DNMT3A* [3, 4, 5, 6]. Our patient developed a very high‐risk MDS with *ASXL1* mutation and trisomy 8 followed by clonal karyotypic evolution after 16 years of well‐controlled HIV infection. Age and time to HIV diagnosis are compatible with what was previously described in HIV/MDS patients [6] and she had no history of classically therapy‐related MDS drugs.

**TABLE 1:** Review of clinical, cytogenetic, and molecular features: Treatment and outcome of well‐controlled HIV‐positive patients with MDS

HIV						MDS						
Case	Age/Sex	Time from HIV (years)	ART	CD4^+^ count (cells/mm^3^)	Viral load	MDS Subtypes	IPSS	Karyotype	Gene mutations	Progression to AML	MDS treatment	Reference^a^
1	63/ M	13	DDC, AZT, 3TC, ABC, EFV, TDF, TFC	296	< 40	RAEB2/AML	>Int 2	Complex with monosomy 7	NA	Yes	Palliative	Rieg et al., 2009 [3]
2	56/F	NA	ABC, 3TC, EFV	206	ND	RAEB	≥Int 2	Complex with del(5q) and monosomy 7	NA	Yes	NA	Takahashi et al., 2012 [4]
3	60/M	NA	ABC, 3TC, LPV	500	NA	RAEB (t‐MDS)	≥Int 2	Complex with del(5q) and monosomy 7	NA	Yes	NA	Takahashi et al., 2012 [4]
4	43/M	NA	NA	223	ND	RAEB	Int‐1	46,XY	NA	No	NA	Takahashi et al., 2012 [4]
5	55/M	23	RTV, DRV, TFC, TDF	500	ND	RA	Int‐2	Complex with monosomy 7	NA	Yes	NA	Takahashi et al., 2012 [4]
6	65/M	26	EFV, TFC, TDF	300	ND	RA	Int‐2	Complex with monosomy 5 and 7	NA	No	NA	Takahashi et al., 2012 [4]
7	50/M	NA	ABC, 3TC, TFC, TDF, LPV	231	NA	RA	Int‐2	46,XY,del (20)(q11)	NA	No	NA	Takahashi et al., 2012 [4]
8	51/M	NA	T20, FTC, TDF, RTV, CRV	254	>75,000	RAEB	Int‐2	47,XY,+ 21	NA	Yes	NA	Takahashi et al., 2012 [4]
9	56/F	NA	ATV, TFC, TDF	1,310	<40	RCMD	Int‐2	del(5q), del(7q), dic(9;12)	NA	Yes	Aza	Williamson et al., 2016 [5]
10	66/F	28	NA	573	52	MDS	Int‐1	46,XX	*ASXL1, DNMT3A*	No	Len, Elt	Kaner et al., 2019 [6]
11	45/M	4	NA	840	ND	t‐ MDS	Int‐2	NA	NA	Yes	Aza	Kaner et al., 2019 [6]
12	66/M	13	NA	383	73	MDS	Int‐2	del(7q)	*ASXL1*	No	Aza	Kaner et al., 2019 [6]
13	70/M	20	NA	76	247	t‐ MDS/ AML	Int‐2	−5, del(7q),−2	*TP53*	Yes	Aza	Kaner et al., 2019 [6]
14	49/M	11	NA	40	ND	MDS/ AML	Int‐2	NA	NA	Yes	Aza	Kaner et al., 2019 [6]
15	54/M	NA	NA	NA	NA	MDS	Int‐2	del(7q)	NA	Yes	Aza, Dec	Kaner et al., 2019 [6]
16	58/F	18	NA	484	ND	MDS	>Int 2	−7	NA	Yes	7+3/ HiDAC	Kaner et al., 2019 [6]
17	66/M	NA	NA	304	ND	t‐ MDS/ AML	Int‐2	del(20q)	*ASXL1*	No	Aza	Kaner et al., 2019 [6]
18	66/F	17	NA	781	ND	MDS	>Int 2	−7, −5, +8, +9	*ASXL1, DNMT3A*,	Yes	Aza	Kaner et al., 2019 [6]
19	52/F	12	NA	140	NA	t‐ MDS/ AML	Int‐2	NA	*TP53*	Yes	Aza	Kaner et al., 2019 [6]
20	56/M	NA	NA	390	ND	MDS	Int‐1	Normal	NA	No	Len, Elt	Kaner et al., 2019 [6]
21	64/F	15	NA	931	<40	MDS/ AML	Int‐2	del(7q)	*ASXL1, TET2, DNMT3A, U2AF1*	Yes	7+3	Kaner et al., 2019 [6]
22	55/M	24	NA	275	<40	t‐ MDS/ AML	>Int 2	Complex with ‐7	*ASXL1, TP53, ETV6*	Yes	7+3/ HiDAC, Dec	Kaner et al., 2019 [6]
23	65/F	2	NA	148	5,599	MDS	Int‐1	del(13q)	ND	No	Palliative	Kaner et al., 2019 [6]
24	61/F	16	AZT, 3TC, EFV	929	ND	MDS‐EB‐1	Int‐2	+8 with clonal karyotypic evolution hyperdiploidy with +8,+10,+21	*ASXL1*	No	Aza	This report

Abbreviations: 3TC, Lamivudine; ABC, Abacavir; AML, Acute myeloid leukemia; ART, Antiretroviral treatment; Aza, Azacitidine; AZT, Zidovudine; Dec, Decitabine, del, deletion; EFV, Efavirenz; Elt, Eltrombopag; F, Female; FTC, Emtricitabine; Int, Intermediate; IPSS, International Prognostic Scoring System; Len, Lenalidomide; LVR, Lopinavir; M, Male; MDS‐EB‐1, myelodysplastic syndrome with excess of blasts 1; NA, Not available; ND, Not detected; RA, Refractory anemia; RAEB, Refractory anemia with excess of blasts; RCMD, Refractory cytopenia with multilineage dysplasia; RTV, Ritonavir; T20, Enfuvirtide; t‐MDS, Therapy‐related myelodysplastic syndrome.

a In this table, the patient collection included 2 case reports and 2 case series, beyond our case.

Hypothetically, MDS development in HIV patients can be related to chronic viral infection, with the inflammatory cytokines environment affecting immune system regulation. ART may also accelerate BM aging leading the acquisition of somatic mutations followed by clonal hematopoiesis [6]. Besides, ART is related to genotoxic effects [11] with genomic instability and loss of heterozygosity [12]. Other previous studies also linked ART to hematopoietic precursor cell dysplastic abnormalities [1, 13, 14]. We hypothesized that the coexistence of trisomy 8 and *ASXL1* mutation in our patient would be a consequence of long‐standing exposition to ART, leading to a sMDS. Mutations in *ASXL1* are detected in 11‐14% of MDS; most of them occur as heterozygous exon 12 frameshift or nonsense mutations and predict inferior prognosis [15]. Isolated trisomy 8 is found in about 7% of MDS cases and is considered a secondary or late event in the MDS evolution [15].

The chromosomes are frequently missegregated during mitosis in cancer cells. This process is known as whole‐chromosome instability (W‐CIN) and leads to aneuploidy. W‐CIN induces tumorigenesis and treatment resistance. The mitotic stress associated with W‐CIN is generally induced by oncogenes and suppressor tumor genes rather than mutations in genes involved in chromosome segregation [16]. Our patient was characterized cytogenetically by an aneuploidy. The *ASXL1* mutation and an extra copy of *c‐myc*, located at 8q24 seem to be possible factors associated with the increase in W‐CIN, leading to clonal evolution and refractoriness to treatment.

Our case report and the literature review highlight the importance of cytogenetic and molecular tests to monitoring HIV‐positive patients under long‐stand treatment and the occurrence of MDS. Often, these patients are not eligible for hematopoietic stem cell transplantation due to their poor clinical condition [6]. Because of the lack of specific treatment for HIV/MDS patients, it is essential to unveil the mechanisms involved in its pathogenesis to led promissory therapy.

## ETHICS APPROVAL AND CONSENT TO PARTICIPATE

Informed consent was obtained from the case in accordance with the Declaration of Helsinki, and ethics was approved by the Ethics and Research Committee of Evandro Chagas Institute for Clinical Research (reference number CAAE #0032.0.009.000‐10).

## CONFLICT OF INTEREST

The authors declare no conflict of interest.

## AUTHOR CONTRIBUTIONS

DPMA and MTS attended the patient, collected and analysed clinical data. TSF, EAAK, and VLL performed cytogenetic and FISH analysis. FVSB, FGA. and VLL performed genetic tests. EPN and BGJG revised the manuscript. MSPO and TSF supervised the study and reviewed the manuscript. MSPO and TSF provided funding. All authors contributed significantly to the work and have seen and approved the manuscript and its submission.

## Data Availability

The documentation is available on a reasonable request to the corresponding author.
